# CBX1 as a Prognostic Biomarker and Therapeutic Target in Liver Hepatocellular Carcinoma: Insight into DNA Methylation and Non-Coding RNA Networks from Comprehensive Bioinformatics Analysis

**DOI:** 10.3390/medicina61060983

**Published:** 2025-05-26

**Authors:** Hye-Ran Kim, Jongwan Kim

**Affiliations:** 1 Department of Biomedical Laboratory Science, Dong-Eui Institute of Technology, 54 Yangji-ro, Busanjin-gu, Busan 47230, Republic of Korea; hrkim@dit.ac.kr; 2 Department of Anatomy, College of Medicine, Dongguk University, Gyeongju 38066, Republic of Korea

**Keywords:** CBX1, LIHC, prognostic biomarker, DNA methylation, non-coding RNAs network

## Abstract

*Background and Objectives*: Chromobox 1 (CBX1), a key epigenetic regulator involved in chromatin remodeling, has been implicated in various cancers; however, its role in liver hepatocellular carcinoma (LIHC) remains underexplored. This study aimed to investigate the expression patterns, epigenetic regulation, and non-coding RNA (ncRNA) networks involving CBX1 in LIHC, assess their potential as diagnostic and prognostic biomarkers, and explore their relevance as a putative therapeutic target. *Materials and Methods*: A multi-omics bioinformatics approach was employed using datasets from GEPIA2, OncoDB, UALCAN, Human Protein Atlas, KM Plotter, MethSurv, miRNet, and ENCORI. These databases were used to analyze mRNA and protein expression, DNA methylation, prognosis, and interaction networks involving CBX1 and ncRNAs. *Results*: CBX1 was significantly upregulated in both the mRNA and protein expression in LIHC. Upregulated CBX1 expression was associated with poor prognosis. DNA methylation analysis revealed that both hypermethylated and hypomethylated probes were significantly associated with CBX1 expression and poor prognosis. hsa-miR-212-3p and hsa-miR-132-3p were significantly upregulated in LIHC and were positively correlated with CBX1 expression and poor prognosis. The ncRNA network was identified, including long ncRNAs, circular RNAs, and pseudogenes, many of which were linked to tumor progression and poor prognosis, and competing endogenous RNAs were associated with tumor progression and poor prognosis in LIHC. *Conclusions*: CBX1 was significantly overexpressed in LIHC and was regulated by both DNA methylation and ncRNA interactions. Its expression is closely associated with a poor prognosis. The CBX1–micro-RNA–long ncRNA/circular RNA axis is a promising avenue for the development of novel diagnostic and therapeutic strategies. This study provides system-level insights into the regulatory landscape of CBX1 in LIHC and supports its potential role in precision medicine.

## 1. Introduction

Liver cancer is currently the sixth most prevalent malignancy and the fourth leading cause of cancer-related mortality worldwide [1]. Liver hepatocellular carcinoma (LIHC) accounts for approximately 80% of primary liver cancers and is a major contributor to cancer-related deaths [2]. Hepatocarcinogenesis involves a range of chronic liver diseases, including hepatitis B and C viral infections, alcoholic liver disease, nonalcoholic steatohepatitis, and nonalcoholic fatty liver disease [3]. Despite recent therapeutic advancements, the prognosis of advanced-stage LIHC remains poor owing to delayed diagnosis, therapeutic resistance, and high recurrence rates [4]. Therefore, there is an urgent need to identify novel biomarkers and therapeutic targets and develop accurate and clinically applicable genetic models for prognosis and precision treatment. Moreover, a variety of blood-based biomarkers, including alpha-fetoprotein (AFP), methylation markers, microRNAs (miRNAs), long non-coding RNAs (lncRNAs), and multi-omics approaches, are being actively investigated for the early detection of LIHC [5].

Epigenetic alterations, particularly DNA methylation, play critical roles in cancer initiation and progression. This occurs via the silencing of tumor suppressor genes through promoter hypermethylation or the activation of oncogenes through hypomethylation [6,7]. Several studies have sought to identify DNA methylation biomarkers relevant to LIHC [8,9,10]. Recent findings have indicated that aberrant DNA methylation is closely associated with LIHC pathogenesis and has potential utility in the discovery of diagnostic and therapeutic biomarkers [11].

Carcinogenesis is a complex multistep process involving both genetic and epigenetic alterations [12]. High-throughput sequencing technologies have identified non-coding RNAs (ncRNAs) as critical regulators of this process [13]. ncRNAs are a diverse group of non-protein-coding RNAs found across species, including long non-coding RNAs (lncRNAs), microRNAs (miRNAs), circular RNAs (circRNAs), pseudogenes, small interfering RNAs, and small nucleolar RNAs. Among these, lncRNAs, miRNAs, pseudogenes, and circRNAs are primarily involved in post-transcriptional gene regulation [14]. Accumulating evidence supports the diagnostic and prognostic potential of ncRNAs in LIHC [15], although their specific biological functions and mechanisms of action remain largely unclear.

Recent studies have highlighted competing endogenous RNA (ceRNA) networks, in which RNAs regulate each other by competing for shared miRNAs at the post-transcriptional level. These networks serve as functional links between coding and ncRNAs, including lncRNAs, circRNAs, miRNAs, and pseudogenes, through shared miRNA response elements, influencing miRNA–mRNA interactions and gene silencing [16]. Dysregulation of ceRNA networks due to abnormal ceRNA expression has been implicated in numerous diseases, including cancer [17].

Similarly, aberrant lncRNA expression has been associated with tumor initiation and progression. In LIHC, lncRNAs have been implicated in tumor angiogenesis, cell proliferation, vascular invasion, and metastasis [18,19,20]. LncRNAs regulate target gene expression and cancer-related signaling pathways, influencing proliferation, migration, invasion, apoptosis, and drug resistance. They can also predict therapeutic outcomes and serve as clinical targets [21].

CircRNAs, another class of ncRNAs, play critical roles in LIHC by acting as miRNA sponges. They are involved in regulating tumor proliferation, immune evasion, metastasis, and drug resistance [22,23,24] and are increasingly being investigated as diagnostic and prognostic biomarkers [25,26].

Pseudogenes, previously regarded as non-functional, have been found to be cancer-specific and abundantly expressed in tumors [27,28]. They may participate in LIHC progression and drug resistance and act as ceRNAs. Their unique expression patterns offer promising avenues for early detection and therapeutic intervention [29].

In this study, we comprehensively analyzed chromobox 1 (CBX1) expression and its regulatory mechanisms in LIHC. Our multi-omics investigation revealed that CBX1 is significantly overexpressed at both the mRNA and protein levels in LIHC and is closely associated with clinicopathological features. Upregulated CBX1 expression was strongly correlated with poor prognosis. DNA methylation analysis demonstrated that both promoter hypermethylation and hypomethylation were significantly associated with CBX1 dysregulation and prognosis. The miRNAs hsa-miR-212-3p and hsa-miR-132-3p were significantly upregulated and positively correlated with CBX1 expression and poor prognosis. These networks revealed extensive interactions of these miRNAs with lncRNAs and circRNAs, which were associated with upregulation and poor prognosis. Pseudogenes were also associated with upregulation and poor prognosis. Correlation analysis revealed that ceRNA genes co-expressed with CBX1 were significantly associated with its upregulation and poor prognosis. Our findings suggest that CBX1 plays a central role in LIHC pathogenesis through complex epigenetic and post-transcriptional regulatory mechanisms involving DNA methylation and ncRNA-mediated networks. CBX1 and its associated ceRNA network may serve as valuable diagnostic and prognostic biomarkers as well as therapeutic targets for precision oncology in LIHC.

## 2. Materials and Methods

### 2.1. mRNA Expression Analysis of CBX1 in LIHC

To evaluate the mRNA expression levels of CBX1 in LIHC, multiple publicly accessible bioinformatics platforms were employed, including GEPIA2 (http://gepia2.cancer-pku.cn/) (accessed on 30 March 2025), OncoDB (https://oncodb.org/) (accessed on 2 April 2025), and UALCAN (http://ualcan.path.uab.edu/ (accessed on 1 April 2025) These databases integrate multi-omics data derived from TCGA to facilitate comprehensive cancer analysis. Differential gene expression between tumor and normal tissues was assessed using GEPIA2, which offers standardized gene expression profiles across various cancer types [30]. OncoDB was used to compare CBX1 expression in tumor and normal tissues across datasets [31]. Additionally, UALCAN provided insights into the expression patterns stratified by clinicopathological subgroups, leveraging its user-friendly interface to enable subgroup-specific analysis [32].

### 2.2. Protein Expression Analysis of CBX1 in LIHC via Immunohistochemistry (IHC)

The protein expression levels of CBX1 in LIHC were validated using IHC data obtained from the Human Protein Atlas (https://www.proteinatlas.org) (accessed on 2 April 2025), which is a comprehensive resource that offers proteomic insights into various cancer types [33,34]. IHC images of the normal liver and LIHC tissues were systematically examined to assess the staining intensity and subcellular localization of CBX1.

### 2.3. Prognostic Analysis of CBX1 in LIHC

To evaluate the prognostic relevance of CBX1 expression in LIHC, Kaplan–Meier (KM) survival analyses were performed using the KM Plotter platform (https://kmplot.com/analysis/) (accessed on 3 April 2025) [35]. The survival endpoints analyzed included overall survival (OS), relapse-free survival (RFS), progression-free survival, and disease-specific survival (DSS).

### 2.4. DNA Methylation and Prognostic Analysis of CBX1 in LIHC

The methylation of CBX1 was assessed using a combination of publicly available bioinformatics platforms, including OncoDB (https://oncodb.org/) (accessed on 2 April 2025) and MethSurv (https://biit.cs.ut.ee/methsurv/) (accessed on 2 April 2025) [36]. OncoDB was used to evaluate the methylation patterns of CBX1 across normal and tumor tissues. Additionally, MethSurv facilitated the analysis of the associations between methylation and patient survival, thereby enabling a comprehensive evaluation of the epigenetic regulation and prognostic significance of CBX1 in LIHC.

### 2.5. Construction of the miRNA–lncRNA–circRNA–mRNA Network and Prognostic Analysis of CBX1of CBX1 in LIHC

To identify candidate miRNAs targeting CBX1, the miRNet database (https://www.mirnet.ca/) (accessed on 4 April 2025) [37] was used. This integrative platform facilitated the prediction of miRNA–mRNA interactions as well as the identification of lncRNA and circRNA interactions associated with the selected miRNAs. To further explore the regulatory network involving CBX1-associated miRNAs, the ENCORI database (https://rnasysu.com/encori/) (accessed on 5 April 2025) [38] was used. ENCORI enabled a comprehensive analysis of miRNA–lncRNA and miRNA–pseudogene correlations specific to LIHC. The expression levels and prognostic significance of CBX1-associated miRNAs, lncRNAs, and pseudogenes were systematically analyzed using the integrated ENCORI. Subsequently, a ceRNA network involving CBX1 was constructed and visualized using the Cytoscape software (version 3.10.2) (http://cytoscape.org/) (accessed on 5 April 2025).

### 2.6. Statistical Analysis

Gene expression data and the corresponding clinical information for tumor and normal tissue samples were obtained from publicly accessible online databases, including GEPIA2, UALCAN, and OncoDB. These platforms integrate gene expression profiles from TCGA and GTEx and provide normalized and curated expression values. The prognostic value of the genes was assessed using KM survival curves and log-rank tests. Survival analyses were conducted using multiple platforms, including KMplot, MethSurv, and ENCORI. Pearson’s correlation analysis was used to evaluate the co-expression relationships between the variables. False discovery rate correction was applied to address the issue of multiple hypothesis testing. Statistical significance was defined as *p* < 0.05 or false discovery rate < 0.05, where applicable.

## 3. Results

### 3.1. mRNA Expression of CBX1 in LIHC

**Figure 1:**
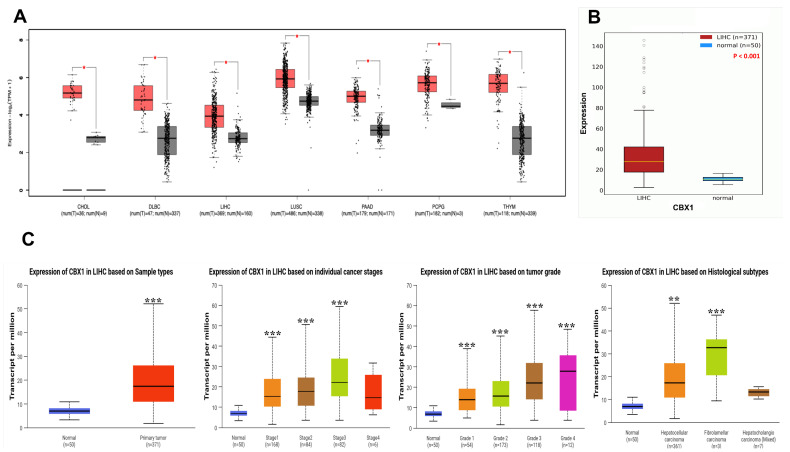
mRNA expression of CBX1 in LIHC. (**A**) Comparison of CBX1 expression between tumor and normal. (**B**) Comparison of CBX1 expression between LIHC and normal. (**C**) Comparison of CBX1 expression between clinicopathologic characteristics and normal. * *p* < 0.05, ** *p* < 0.01, and *** *p* < 0.001.

Differential expression of CBX1 between tumor and normal tissues from various cancer types, including LIHC, was analyzed using the GEPIA2 database. The results demonstrated a marked upregulation of CBX1 expression in LIHC as well as in various cancers, such as cholangiocarcinoma, diffuse large B-cell lymphoma, lung squamous cell carcinoma, pancreatic adenocarcinoma, pheochromocytoma, paraganglioma, and thymoma (Figure 1A). To further validate the findings specific to LIHC, the OncoDB database was used, which confirmed the significant overexpression of CBX1 in LIHC tissues compared with normal tissues (Figure 1B). We also examined the association between CBX1 expression and various clinicopathological features of LIHC. The analysis revealed statistically significant correlations between CBX1 expression and primary tumor, tumor stage (I–III), tumor grade (I–IV), and histological subtype (Figure 1C). These findings suggest that CBX1 is significantly upregulated in LIHC and may be associated with tumor progression and distinct pathological features.

### 3.2. Protein Expression of CBX1 in LIHC

**Figure 2:**
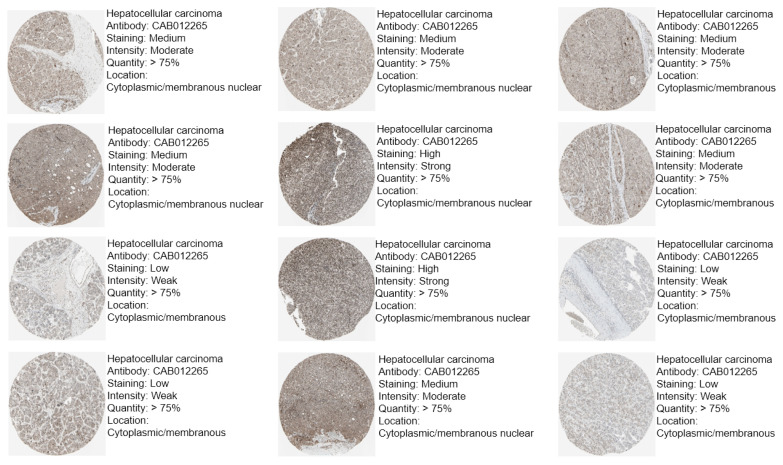
Protein expression of CBX1 in immunohistochemical images of LIHC.

To assess the protein expression of CBX1 in LIHC, data from the Human Protein Atlas database were analyzed. The results revealed a significant upregulation of CBX1 protein expression in LIHC tissues compared with normal tissues (Figure 2). Collectively, these findings indicate that CBX1 is overexpressed in LIHC at both the transcriptional and translational levels, suggesting a potential role in hepatocarcinogenesis.

### 3.3. Prognostic Value of CBX1 Expression in LIHC

**Figure 3:**
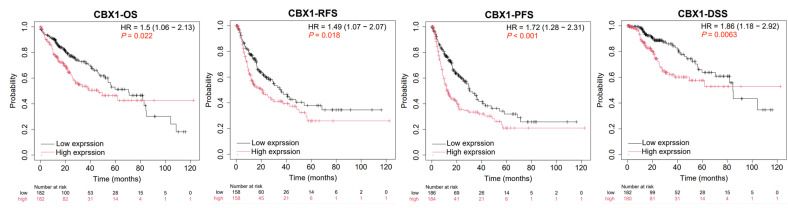
Prognostic significance of CBX1 expression in LIHC. OS, overall survival; RFS, relapse-free survival; PFS, progression-free survival; DSS, disease-free survival.

To assess the prognostic significance of CBX1 expression in LIHC, KM survival analyses were conducted using the KM database. The survival endpoints evaluated were OS, RFS, PFS, and DSS. Elevated CBX1 expression was significantly associated with worse prognosis, including OS (hazard ratio (HR) = 1.5, *p* = 0.022), RFS (HR = 1.49, *p* = 0.018), progression-free interval (HR = 1.72, *p* < 0.001), and DSS (HR = 1.86, *p* = 0.0063) (Figure 3). These findings suggest that high CBX1 expression is a potential indicator of poor prognosis in patients with LIHC.

### 3.4. Relationship Between CBX1 Expression Level and Clinicopathological Characteristics of LIHC

We investigated the correlation between CBX1 expression and the clinicopathological characteristics of LIHC. Higher CBX1 expression was significantly associated with poorer survival outcomes across multiple clinicopathological subgroups. For OS, elevated CBX1 expression correlated with worse prognosis in patients with grade II (HR = 1.99, *p* = 0.0094), asian patients (HR = 2.99, *p* = 0.00052), alcohol consumption (HR = 1.91, *p* = 0.043), and no hepatitis virus infection (HR = 2.79, *p* = 0.000037). In terms of RFS, significantly poorer outcomes were observed in females (HR = 1.84, *p* = 0.044), grade II patients (HR = 1.84, *p* = 0.014), Asian patients (HR = 1.74, *p* = 0.032), sorafenib treatment (HR = 3.34, *p* = 0.023), alcohol consumption (HR = 2.26, *p* = 0.0063), and no hepatitis virus infection (HR = 2.16, *p* = 0.003). For PFS, CBX1 overexpression was correlated with poorer outcomes in males (HR = 1.79, *p* = 0.0014), females (HR = 1.71, *p* = 0.04), grade II (HR = 2.38, *p* = 0.000097), white patients (HR = 1.85, *p* = 0.0021), Asian patients (HR = 1.97, *p* = 0.0047), alcohol consumption (HR = 2.65, *p* = 0.00032), and no hepatitis virus infection (HR = 2.65, *p* = 0.00023). Regarding disease-specific survival (DSS), significant associations were found in males (HR = 1.76, *p* = 0.05), females (HR = 2.21, *p* = 0.04), grade II (HR = 2.92, *p* = 0.0018), white patients (HR = 2.05, *p* = 0.013), Asian patients (HR = 3.56, *p* = 0.0024), alcohol consumption (HR = 2.29, *p* = 0.023), and no hepatitis virus infection (HR = 4.22, *p* = 0.0000057). These results collectively suggest that CBX1 overexpression is strongly correlated with poor prognosis in LIHC, especially among specific clinical subgroups (Table 1).

### 3.5. Correlation of CBX1 Expression with DNA Methylation in LIHC

**Figure 4:**
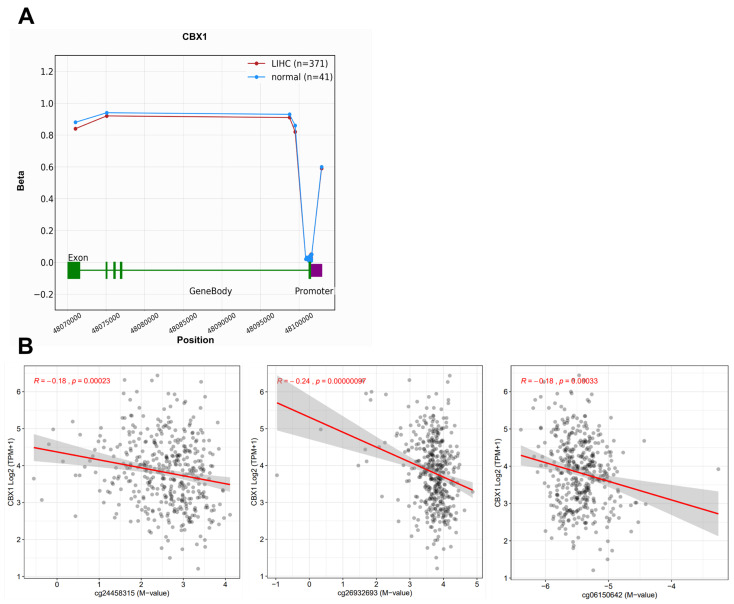
DNA methylation of CBX1 expression in LIHC. (**A**) DNA methylation of CBX1 between LIHC and normal. (**B**) Correlation between DNA methylation and CBX1 expression in LIHC.

**Figure 5:**
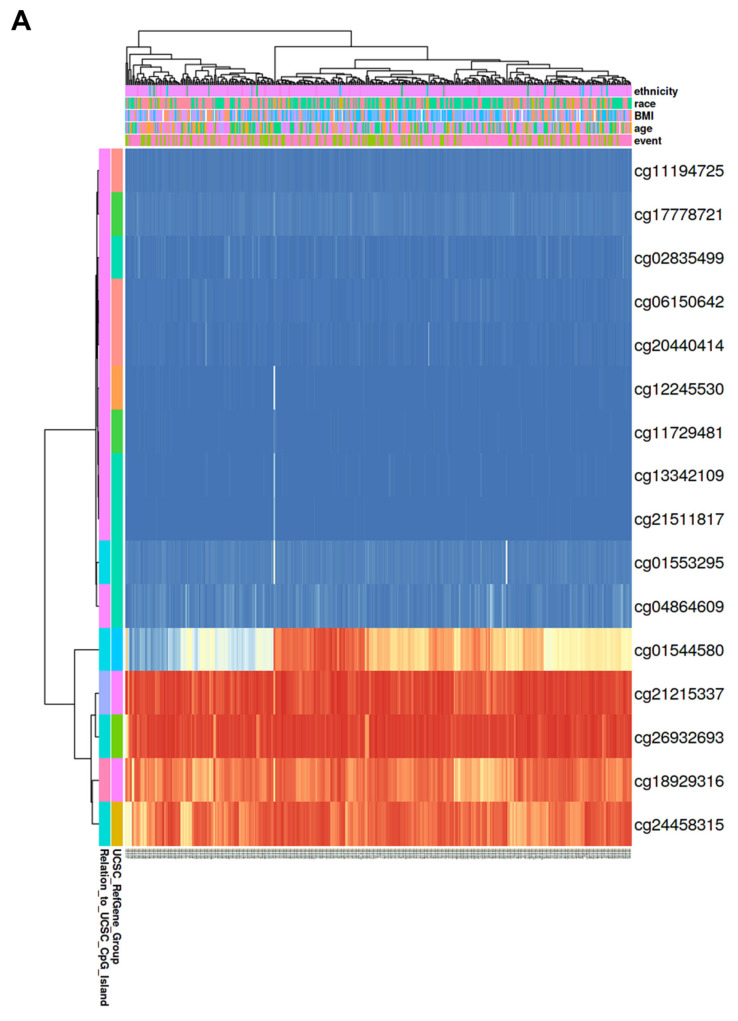

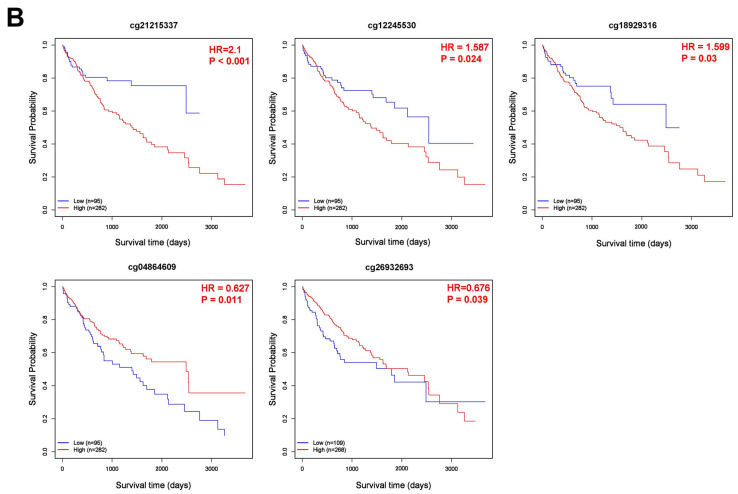
Prognostic significance of DNA methylation of CBX1 in LIHC. (**A**) Heatmap of DNA methylation of CBX1. (**B**) KM plotter of DNA methylation of CBX1.

Analyses were performed using the OncoDB database to explore the potential regulatory role of DNA methylation in CBX1 expression in LIHC. CBX1 expression was significantly correlated with DNA methylation at specific probes located within both the promoter and exon regions in LIHC and normal tissues (Figure 4 and Table 2). The results reveal that nine sites demonstrated statistically significant differences in methylation levels. The results reveal that hypomethylation was observed at several sites, including cg24458315 (*p* < 0.001), cg26932693 (*p* = 0.0012), cg21215337 (*p* < 0.001), and cg18929316 (*p* < 0.001). Conversely, hypermethylation was observed at several sites, including cg04864609 (*p* < 0.001), cg02835499 (*p* < 0.001), cg06150642 (*p* = 0.0025), cg11194725 (*p* = 0.014), and cg21511817 (*p* = 0.0076). Among these, three probes exhibited a statistically significant negative correlation with the expression of CBX1. These are cg24458315 (R = −0.18, *p* < 0.001), cg26932693 (R = −0.24, *p* < 0.001), and cg06150642, (R = −0.18, *p* < 0.001). In the generated heat map, hypermethylated regions are shown in red, whereas hypomethylated regions are shown in blue (Figure 5A). To determine the prognostic significance of CBX1 methylation-associated probes in LIHC, survival analysis was conducted using the MethSurv database. The findings revealed that hypermethylated probes, including cg21215337 (HR = 2.1, *p* < 0.001), cg12245530 (HR = 1.587, *p* = 0.024), and cg18929316 (HR = 1.599, *p* = 0.03), were significantly associated with poor prognosis in LIHC. Hypomethylated probes, such as cg04864609 (HR = 0.627, *p* = 0.011) and cg26932693 (HR = 0.676, *p* = 0.039), were also associated with poor prognosis in LIHC (Figure 5B). These results suggest that epigenetic alterations involving both promoter and exon methylation may contribute to the dysregulation of CBX1 expression and are potentially implicated in the poor prognosis of patients with LIHC.

### 3.6. Prediction of Target miRNAs and Construction of the CBX1-Associated Co-Expression Network

**Figure 6:**
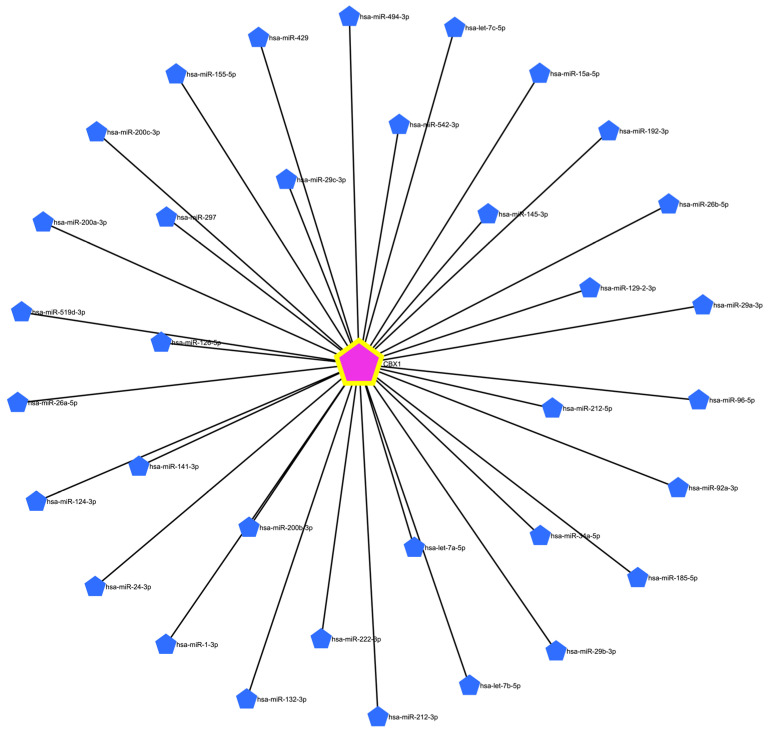
miRNA network associated with CBX1.

**Figure 7:**
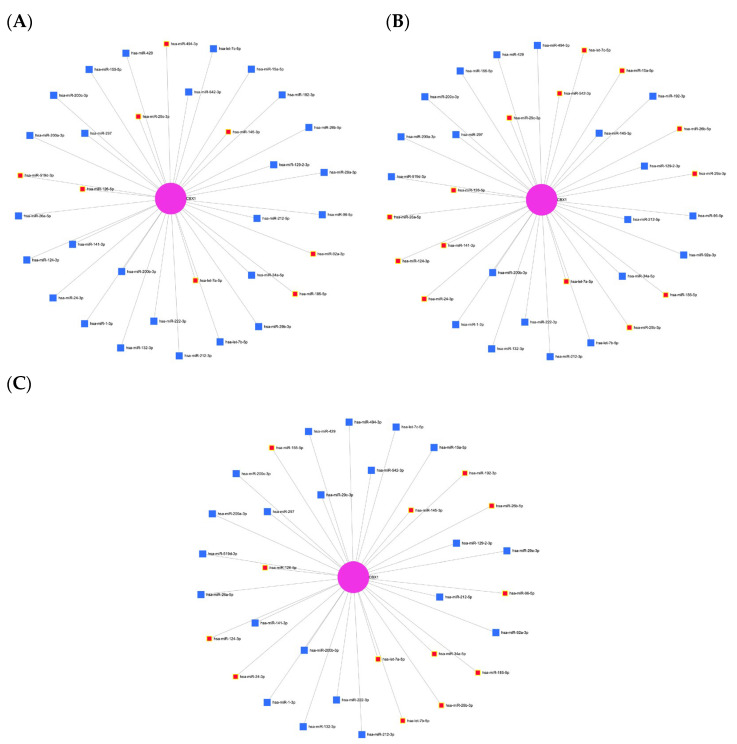
CBX1-associated miRNA network in liver. (**A**) miRNA network associated with CBX1 in liver cirrhosis. (**B**) miRNA network associated with CBX1 in hepatitis B virus infection. (**C**) miRNA network associated with CBX1 in hepatitis C virus infection.

**Figure 8:**
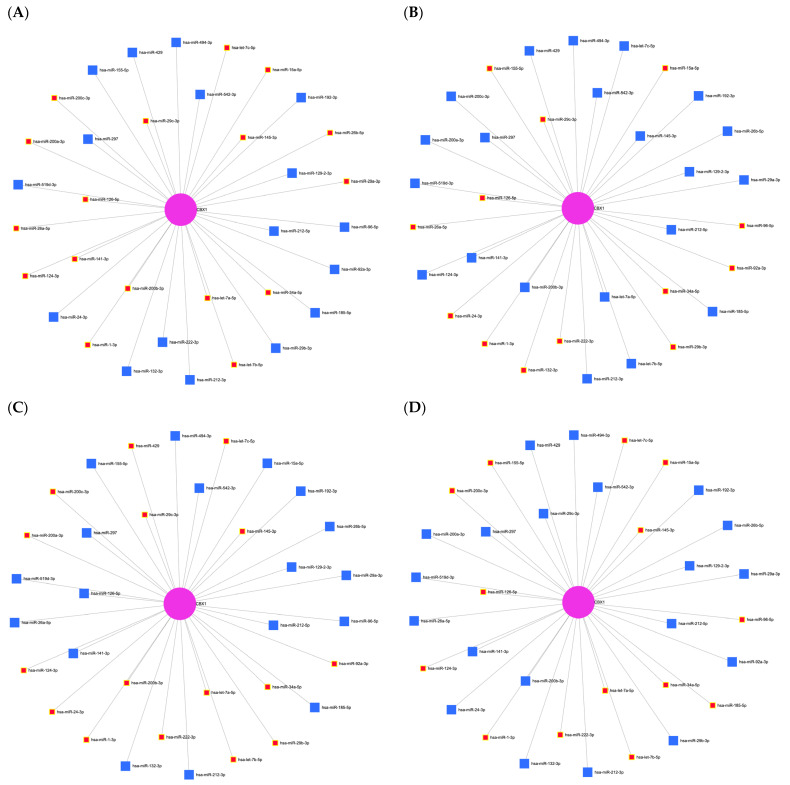
Function network of CBX1-associated miRNA. (**A**) miRNA network associated with CBX1 in tumor-suppressive activity. (**B**) miRNA network associated with CBX1 in apoptosis. (**C**) miRNA network associated with CBX1 in cell proliferation. (**D**) miRNA network associated with CBX1 in cell differentiation.

To identify potential miRNAs targeting CBX1, a predictive analysis was conducted using the miRNet database, a comprehensive platform for investigating miRNA-target interactions. This analysis identified 34 miRNAs that were potentially associated with CBX1 (Figure 6 and Table 3). To confirm these findings, we explored the involvement of these miRNAs in liver-associated diseases. Several CBX1-related miRNAs have been implicated in liver cirrhosis, including hsa-miR-494-3p, hsa-miR-29c-3p, hsa-miR-145-3p, hsa-miR-159d-3p, hsa-miR-126-5p, hsa-miR-92a-3p, hsa-miR-185-5p, and hsa-let-7a-5p (Figure 7A). Additionally, miRNAs such as hsa-miR-15a-5p, hsa-miR-542-3p, hsa-miR-29c-3p, hsa-miR-141-3p, hsa-miR-124-3p, hsa-miR-24-3p, hsa-miR-29b-3p, hsa-miR-29a-3p, hsa-miR-26a-5p, hsa-miR-26-5p, hsa-miR-185-5p, hsa-let-7c-5p, and hsa-let-7a-5p were found to be associated with hepatitis B virus infection (Figure 7B), while hsa-miR-192-3p, hsa-miR-145-3p, hsa-miR-29b-3p, hsa-miR-24-3p, hsa-miR-124-3p, hsa-miR-155-5p, hsa-miR-26b-5p, hsa-miR-34a-5p, hsa-miR-185-5p, hsa-miR-126-5p, hsa-let-7a-5p, and hsa-let-7b-5p were linked to hepatitis C virus infection (Figure 7C). To assess the biological relevance of these miRNAs, we performed functional annotation focusing on the processes of cancer biology. Several miRNAs (hsa-miR-145-3p, hsa-miR-29a-3p, hsa-miR-1-3p, hsa-miR-200b-3p, hsa-miR-124-3p, hsa-miR-141-3p, hsa-miR-200a-3p, hsa-miR-200c-3p, hsa-miR-29c-3p, hsa-miR-15a-5p, hsa-miR-34a-5p, hsa-miR-126-5p, hsa-let-7a-5p, hsa-let-7b-5p, and hsa-let-7c-5p) were associated with tumor-suppressive activity (Figure 8A). Additionally, several miRNAs were identified, including hsa-miR-29c-3p, hsa-miR-92a-3p, hsa-miR-29b-3p, hsa-miR-222-3p, hsa-miR-132-3p, hsa-miR-1-3p, hsa-miR-24-3p, hsa-miR-155-5p, hsa-miR-15a-5p, hsa-miR-96-5p, and hsa-miR-34a-5p (Figure 8B). Moreover, several miRNAs, including hsa-miR-145-3p, hsa-miR-92a-3p, hsa-miR-29b-3p, hsa-miR-29c-3p, hsa-miR-222-3p, hsa-miR-200b-3p, hsa-miR-1-3p, hsa-miR-24-3p, hsa-miR-124-3p, hsa-miR-200a-3p, hsa-miR-200c-3p, hsa-miR-34a-5p, hsa-let-7a-5p, hsa-let-7c-5p, and hsa-miR-429 were implicated in the regulation of cell proliferation (Figure 8C). Moreover, several miRNAs, such as hsa-miR-145-3p, hsa-miR-222-3p, hsa-miR-1-3p, hsa-miR-124-3p, hsa-miR-200c-3p, hsa-miR-96-5p, hsa-miR-185-5p, hsa-miR-155-5p, hsa-let-7a-5p, hsa-let-7b-5p, and hsa-let-7c-5p, were found to be involved in cell differentiation (Figure 8D). These findings suggest that a complex regulatory network of CBX1-associated miRNAs is involved in the pathogenesis of liver disease and key cancer-related processes, underscoring their potential significance as biomarkers and therapeutic targets in LIHC.

### 3.7. Expression and Prognostic Significance of CBX1-Associated miRNAs in LIHC

**Figure 9:**
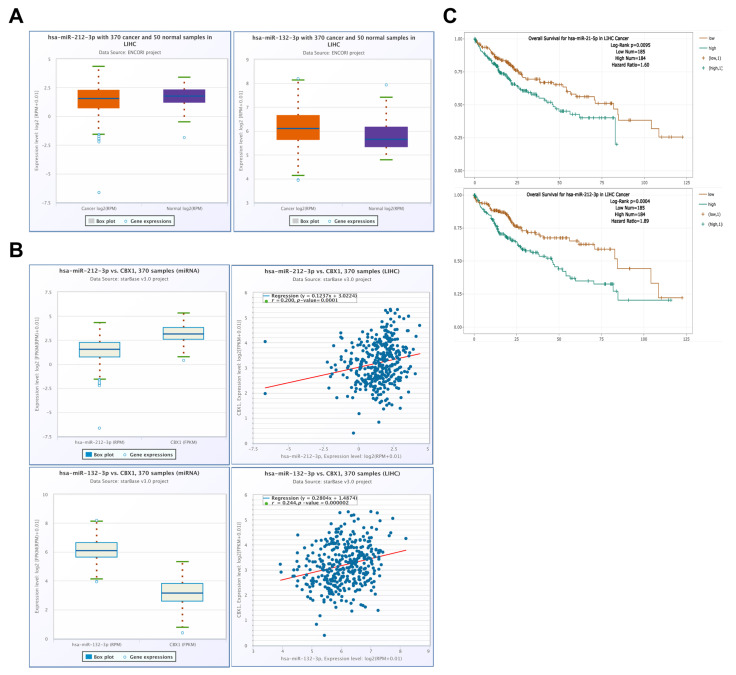
Expression and prognostic significance of CBX1-associated miRNAs in LIHC. (**A**) Expression of hsa-miR-212-3p and hsa-miR-132-3p in LIHC compared with normal. (**B**) Expression and correlation between CBX1 and hsa-miR-212-3p and hsa-miR-132-3p in LIHC. (**C**) KM plotter of hsa-miR-212-3p and hsa-miR-132-3p in LIHC.

To assess the expression and potential prognostic value of CBX1-targeting miRNAs in LIHC, data from the ENCORI database were systematically analyzed. Among the predicted candidates, hsa-miR-212-3p and hsa-miR-132-3p were significantly upregulated in LIHC tissues compared with normal tissues (Figure 9A). The hsa-miR-212-3p expression was lower than that of CBX1, demonstrating a significant positive correlation with CBX1 expression in LIHC (R = 0.200, *p* < 0.001). Similarly, hsa-miR-132-3p showed upregulated expression and a positive correlation with CBX1 expression in LIHC (R = 0.244, *p* < 0.001; Figure 9B). Survival analysis revealed that higher expression levels of both hsa-miR-212-3p and hsa-miR-132-3p were significantly associated with a poorer prognosis in LIHC (Figure 9C). These findings suggested that both hsa-miR-212-3p and hsa-miR-132-3p may serve as potential prognostic indicators and play functional roles in the regulatory network involving CBX1 in LIHC.

### 3.8. Construction of lncRNA and circRNA Networks for CBX1-Associated miRNAs in LIHC

**Figure 10:**
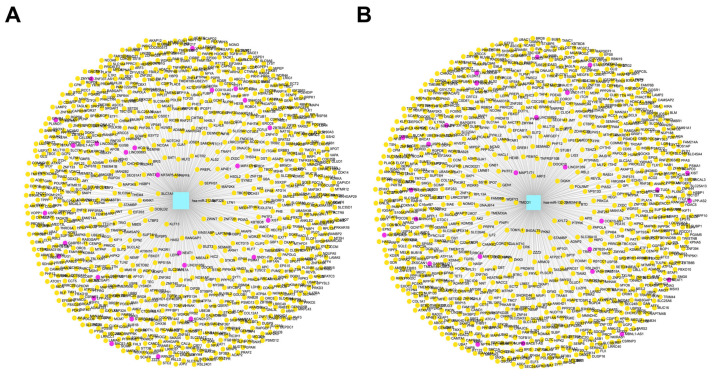
lncRNA and circRNA interaction networks of CBX1-associated miRNA. (**A**) lncRNA and circRNA interaction networks of hsa-miR-212-3p. (**B**) lncRNA and circRNA interaction networks of hsa-miR-132-3p.

To elucidate the regulatory network involving CBX1-associated miRNAs in LIHC, interaction analyses were conducted using the miRNet database to construct lncRNA and circRNA networks. This study focused on two key miRNAs, hsa-miR-212-3p and hsa-miR-132-3p, previously identified as potential post-transcriptional regulators of CBX1. The analysis revealed that lncRNAs associated with hsa-miR-212-3p were predicted to interact with 28 target genes (red), whereas circRNAs linked to the same miRNA interacted with 1323 target genes (yellow) (Figure 10A, Supplementary Table S1). Similarly, lncRNAs associated with hsa-miR-132-3p were found to target 28 genes (red) and the corresponding circRNAs were predicted to interact with 1321 genes (yellow) (Figure 10B, Supplementary Table S1). These findings suggest that lncRNA- and circRNA-mediated networks play a substantial role in modulating the function and regulatory impact of CBX1-associated miRNAs in LIHC pathogenesis.

### 3.9. Correlation of lncRNA Genes Associated with CBX1-Targeting miRNAs in LIHC

**Figure 11:**
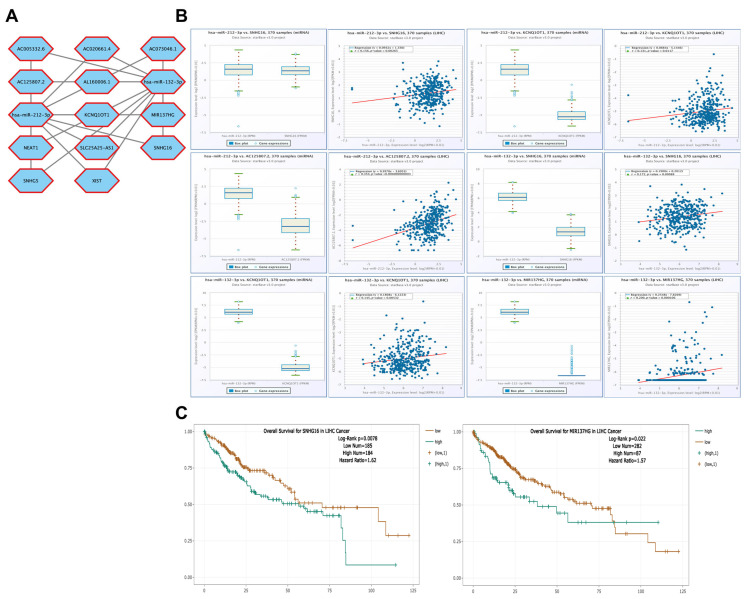
lncRNA interaction networks and prognostic significance of CBX1-associated miRNAs in LIHC. (**A**) lncRNA interaction networks of hsa-miR-212-3p and hsa-miR-132-3p. (**B**) Expression and correlation of hsa-miR-212-3p and hsa-miR-132-3p. (**C**) KM plotter of IncRNA.

Correlation analyses were performed using the ENCORI database to investigate the potential regulatory relationships between lncRNAs and CBX1-targeting miRNAs in LIHC. The analysis focused on two miRNAs, hsa-miR-212-3p and hsa-miR-132-3p, which were previously shown to be upregulated in LIHC. The miRNAs hsa-miR-212-3p and hsa-miR-132-3p interacted with 12 lncRNAs (Figure 11A). High hsa-miR-212-3p expression was positively correlated with SNHG16 (R = 0.156, *p* = 0.00265), KCNQ1OT1 (R = 0.131, *p* = 0.0117), and AC125807.2 (R = 0.353, *p* < 0.001). Similarly, high hsa-miR-132-3p expression was positively correlated with SNHG16 (R = 0.172, *p* < 0.001), KCNQ1OT1 (R = 0.145, *p* = 0.00532), and MIR137HG (R = 0.200, *p* < 0.001) (Figure 11B). Furthermore, survival analysis revealed that elevated expression of SNHG16 (HR = 1.62, *p* = 0.0078) and MIR137HG (HR = 1.57, *p* = 0.022) was significantly associated with poor prognosis of LIHC (Figure 11C). These findings suggest that lncRNAs correlated with these two miRNAs may play important roles in the regulation of CBX1 expression and the progression of LIHC.

### 3.10. Correlation of Pseudogenes Associated with CBX1-Targeting miRNAs in LIHC

**Figure 12:**
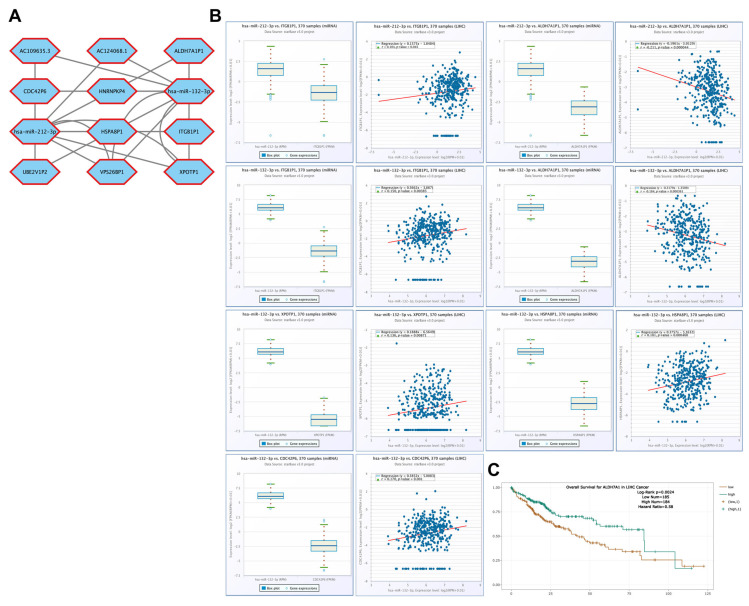
Pseudogene interaction networks and prognostic significance of CBX1-associated miRNA in LIHC. (**A**) Pseudogene interaction networks of hsa-miR-212-3p and hsa-miR-132-3p. (**B**) Expression and correlation of hsa-miR-212-3p and hsa-miR-132-3p. (**C**) KM plot of pseudogene.

Correlation analyses were performed using the ENCORI database to examine the potential involvement of pseudogenes in regulatory networks of CBX1-targeting miRNAs in LIHC. The miRNAs hsa-miR-212-3p and hsa-miR-132-3p interacted with 10 lncRNAs (Figure 12A). High hsa-miR-212-3p expression was positively correlated with ITGB1P1 (R = 0.104, *p* = 0.045), and it was negatively correlated with ADH7A1P1 (R = –0.211, *p* < 0.001). Additionally, high hsa-miR-132-3p expression was positively correlated with ITGB1P1 (R = 0.150, *p* = 0.00385), XPOTP1 (R = 0.136, *p* = 0.00871), HSPA8P1 (R = 0.181, *p* < 0.001), and CDC42P6 (R = 0.170, *p* = 0.001). In contrast, high hsa-miR-212-3p expression was negatively correlated with ADH7A1P1 (R = –0.184, *p* < 0.001). Furthermore, survival analysis indicated that high ADH7A1P1 (HR = 0.58, *p* = 0.0024) expression was significantly associated with poor prognosis in LIHC (Figure 12C). These findings underscore the potential role of pseudogenes as components of the CBX1-associated miRNA regulatory network and suggest their possible contribution to LIHC progression.

### 3.11. Correlation of ceRNAs Associated with CBX1 in LIHC

**Figure 13:**
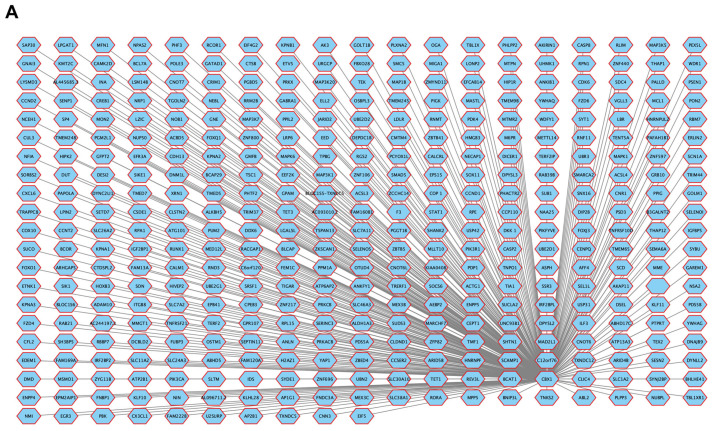

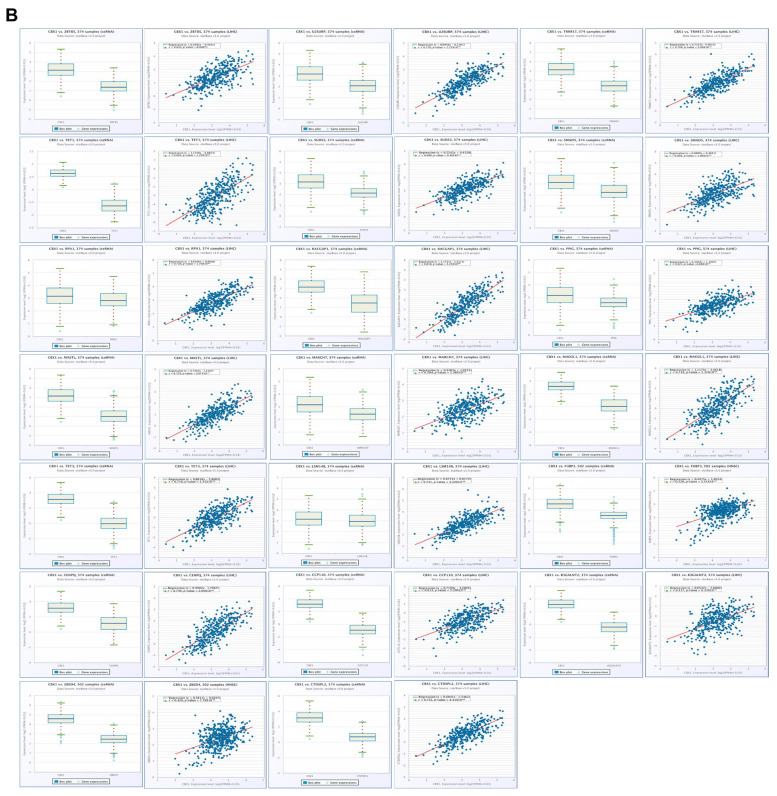

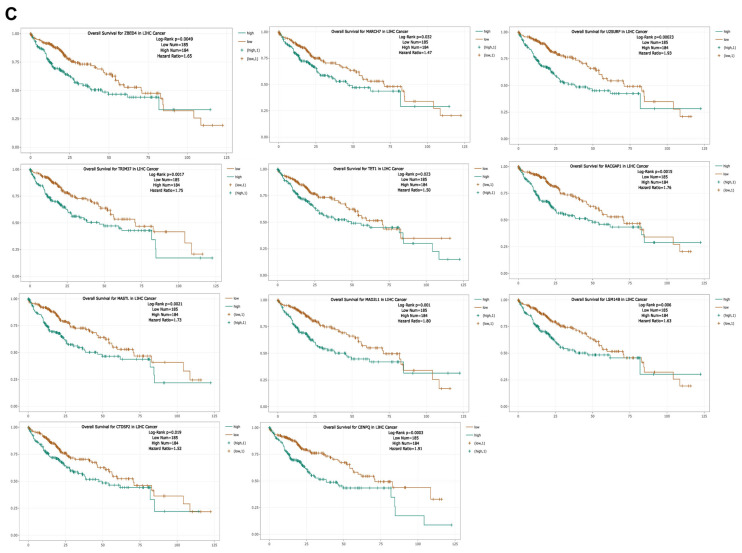
CeRNA interaction networks and prognostic significance of CBX1. (**A**) CeRNA interaction networks of CBX1. (**B**) Expression and correlation of CBX1-associated ceRNAs. (**C**) KM plot of CBX1-associated ceRNAs.

Correlation analyses were performed using the ENCORI database to explore the involvement of ceRNAs in the regulation of CBX1 in LIHC. A total of 389 ceRNAs were identified as potential regulators of CBX1 expression (Figure 13A, Supplementary Table S2). To refine the analysis, we focused on the top 20 ceRNAs that exhibited the strongest correlation with CBX1 expression in LIHC. The results revealed that the expression of these ceRNA genes was significantly upregulated and positively correlated with that of CBX1. Notable correlations were observed with ZBTB5 (R = 0.625, *p* < 0.001), U25URP (R = 0.776, *p* < 0.001), TRIM37 (R = 0.766, *p* < 0.001), TET1 (R = 0.663, *p* < 0.001), SUDS3 (R = 0.688, *p* < 0.001), SMAD5 (R = 0.603, *p* < 0.001), RPA1 (R = 0.726, *p* < 0.001), RACGAP1 (R = 0.810, *p* < 0.001), PPIG (R = 0.621, *p* < 0.001), MASTL (R = 0.759, *p* < 0.001), MARCH7 (R = 0.584, *p* < 0.001), MAD2L1 (R = 0.716, *p* < 0.001), TET3 (R = 0.710, *p* < 0.001), LSM14B (R = 0.624, *p* < 0.001), FUBP3 (R = 0.528, *p* < 0.001), CENPQ (R = 0.730, *p* < 0.001), CCP110 (R = 0.613, *p* < 0.001), B3GALNT2 (R = 0.517, *p* < 0.001), ZBED4 (R = 0.428, *p* < 0.001), and CTDSPL2 (R = 0.732, *p* < 0.001) (Figure 13B). Subsequent survival analysis indicated that the high expression of several of these ceRNAs was significantly associated with poor prognosis in LIHC. These included ZBED4 (HR = 1.65, *p* = 0.0049), MARCH7 (HR = 1.47, *p* = 0.032), U25URP (HR = 1.93, *p* < 0.001), TRIM37 (HR = 1.75, *p* = 0.0017), TET1 (HR = 1.50, *p* = 0.023), RACGAP1 (HR = 1.76, *p* = 0.0015), MASTL (HR = 1.73, *p* = 0.0021), MAD2L1 (HR = 1.80, *p* = 0.001), LSM14B (HR = 1.63, *p* = 0.006), CTDSP2 (HR = 1.52, *p* = 0.019), and CENPQ (HR = 1.91, *p* < 0.001) (Figure 13C). These findings suggest that CBX1 participates in a ceRNA regulatory network that plays a role in tumor progression and prognosis in LIHC.

## 4. Discussion

Liver cancer is among the most fatal malignancies worldwide, ranking as the sixth most diagnosed cancer and fourth leading cause of cancer-related mortality [1]. LIHC accounts for approximately 80% of all primary liver cancers and continues to impose a significant clinical burden [2]. The pathogenesis of LIHC is multifactorial, with well-established risk factors, including chronic liver diseases caused by hepatitis B virus, hepatitis C virus, alcohol consumption, and nonalcoholic fatty liver disease [39]. These factors may induce DNA damage, epigenetic alterations, and cancer-related mutations, leading to the silencing of tumor suppressors and activation of oncogenes, which eventually contribute to LIHC progression [40,41]. Several preventive and therapeutic strategies have been implicated in the management of LIHC, as exampled by the administration of anti-hepatitis vaccine, specific kinase inhibitors, surgical resection, and liver transplantation [42,43] These treatments, together with biomarker screening (e.g., a-fetoprotein), have minimized LIHC-related death to a certain extent, but their performance is far from acceptable therapeutic effect, thus novel diagnostic/therapeutic approaches are still urgently needed to improve the clinic outcomes of LIHC [44]

DNA methylation plays a pivotal role in the initiation and progression of cancer by mediating the epigenetic silencing of tumor suppressor genes through promoter hypermethylation and facilitating oncogene activation via promoter hypomethylation. Cancer-associated alterations in DNA methylation patterns frequently emerge during the early stages of carcinogenesis, often preceding morphological transformation [45]. Aberrant DNA methylation is widely recognized as a hallmark of cancer; however, its specific contribution to tumorigenesis and clinical prognosis remains incompletely understood [46]. Consequently, DNA methylation has considerable potential as a prognostic and diagnostic biomarker. Although numerous potential biomarkers for LIHC have recently been identified, few DNA methylation markers have been clinically validated for this malignancy [9,47]. Our methylation analysis revealed that both promoter and exon regions of the CBX1 gene exhibit aberrant methylation patterns significantly correlated with gene expression and prognosis in LIHC. Notably, several hyper- and hypomethylated probes were associated with poor survival outcomes, suggesting that epigenetic dysregulation of CBX1 may contribute to tumor progression and serve as a prognostic indicator in LIHC.

LncRNAs are increasingly recognized as key regulators of a wide range of physiological and pathological processes. These non-coding transcripts exhibit cancer-specific expression patterns and have been shown to influence tumor growth, survival, and progression across various malignancies [48]. Under normal physiological conditions, lncRNAs contribute to maintaining liver homeostasis by modulating immune responses, supporting tissue regeneration, and preserving the hepatic microenvironment. However, sustained proliferative signaling potentially driven by dysregulated lncRNA expression may contribute to hepatocarcinogenesis. Aberrant transcriptional activity or post-transcriptional modifications may lead to the overexpression of oncogenic lncRNAs or the suppression of tumor-suppressive lncRNAs, thereby contributing to pathological conditions such as chronic hepatitis, hepatomegaly, and oxidative stress, all of which are implicated in the initiation and progression of LIHC [20]. In LIHC, dysregulated expression of lncRNAs has been confirmed to exert both tumor-promoting and tumor-suppressive effects. These molecules are thought to be involved in critical biological processes, including cellular proliferation, migration, invasion, apoptosis, and resistance to therapy, ultimately influencing liver cancer progression [49,50]. Moreover, lncRNAs are being actively investigated as predictive biomarkers of therapeutic efficacy and as potential targets for precision oncology [21]. Our analysis revealed that CBX1-associated miRNAs, particularly hsa-miR-212-3p and hsa-miR-132-3p, engage in complex interactions with numerous lncRNAs and circRNAs. This suggests that lncRNA- and circRNA-based ceRNA networks may regulate CBX1 expression and significantly contribute to the pathogenesis of LIHC.

circRNAs have emerged as critical regulatory molecules implicated in the pathogenesis of numerous human diseases, including cardiovascular disorders, diabetes, neurological conditions, and various cancers [51,52]. Growing evidence suggests that circRNAs play pivotal roles in LIHC initiation and progression. CircRNAs function as oncogenic regulators in LIHC. Previous studies have reported that circ-0008450 has been shown to enhance the proliferation, invasion, and migration of LIHC cells, while inhibiting apoptosis by modulating miR-548p [53]. Similarly, circRNA-104718 promotes the proliferation and invasion of LIHC cells by regulating miRNA-218-5p [54]. Conversely, some circRNAs exhibit tumor-suppressive properties. For example, circADAMTS14 suppresses hepatocellular carcinoma (HCC) cell proliferation, invasion, and migration while promoting apoptosis via the miR-572/RCAN1 axis [55]. Similarly, circRNA-5692 exerted inhibitory effects on LIHC progression by regulating the miR-328-5p/DAB2IP pathway [56]. Despite these observations, the functional roles of circRNAs in LIHC remain largely speculative, and further experimental studies are required to delineate their mechanistic contributions to hepatocarcinogenesis.

Pseudogenes are frequently and abundantly expressed in tumor tissues, and in many instances, their expression is restricted to malignant regions, underscoring their potential utility as cancer-specific biomarkers [28]. Pseudogenes have been demonstrated to exert both oncogenic and tumor-suppressive functions in various malignancies, including LIHC [57,58]. The growing recognition of their functional significance has contributed to a deeper understanding of the molecular heterogeneity of LIHC and presents new opportunities for the development of innovative diagnostic biomarkers and targeted therapeutic strategies. Although numerous pseudogenes have been found to be aberrantly expressed and implicated in LIHC tumorigenesis [59,60], relatively few have been systematically characterized in the context of early recurrence. Further research is essential to elucidate the clinical relevance and mechanistic roles of these pseudogenes in LIHC progression and recurrence.

ceRNAs represent a class of ncRNAs capable of competitively binding to shared miRNAs, thereby facilitating cross-regulation at the post-transcriptional level. However, ceRNA regulatory networks have been suggested to play roles in processes such as cellular proliferation, metastasis, epithelial-to-mesenchymal transition, and chemoresistance in LIHC [61,62,63]. These ceRNA networks offer novel insights for the diagnosis and treatment of HCC. Despite the identification of several ceRNAs involved in HCC progression [64,65,66], our current understanding of their regulatory roles in HCC remains limited. Further investigations are warranted to elucidate the unexplored functions and underlying mechanisms of ceRNA-mediated regulation in this malignancy. In addition to its involvement in epigenetic and post-transcriptional regulatory networks, CBX1 also plays a mechanistic role in chromatin remodeling. As a member of the heterochromatin protein 1 (HP1) family, CBX1 contributes to the formation and maintenance of heterochromatin, thereby promoting transcriptional repression [67]. This function is critical for regulating gene expression during tumorigenesis, and its chromatin-related activities further support the potential of CBX1 as a biomarker in LIHC.

## 5. Conclusions

This study comprehensively elucidates the oncogenic role and regulatory network of CBX1 in LIHC cells. CBX1 was significantly overexpressed in LIHC at both the mRNA and protein levels and correlated with clinicopathological features and poor prognosis. Epigenetic analysis revealed that DNA methylation at specific promoter and exon sites may contribute to the dysregulation of CBX1 expression and its prognostic implications. Moreover, we identified a set of CBX1-targeting miRNAs, including hsa-miR-212-3p and hsa-miR-132-3p, which were upregulated in LIHC and associated with poor prognosis. These miRNAs were integrated into ncRNA networks that include lncRNAs, circRNAs, and pseudogenes. Bioinformatic tools were used to analyze and discuss the crucial pathways, diagnostic performance, and prognostic value of key genes. The lncRNAs, circRNAs, miRNAs, and targeted mRNAs involved in the ceRNA network may be potential diagnostic biomarkers and therapeutic targets for LIHC. Collectively, our findings underscore the multifaceted contribution of CBX1 to LIHC pathogenesis through transcriptional, epigenetic, and post-transcriptional regulation. Further mechanistic and experimental studies are warranted to validate the clinical utility of CBX1 and its regulatory networks as novel targets for diagnosis and prognosis intervention in LIHC.

## Figures and Tables

**Table 1:** Relationship between CBX1 expression level and clinicopathological characteristics of LIHC.

Clinicopathological Characteristics	Overall Survival (*n* = 3218)			Relapse Free Survival (*n* = 2809)			Progression Free Survival (*n* = 3162)			Disease Specific Survival (*n* = 3189)		
	N	Hazard Ratio	*p*-Value	N	Hazard Ratio	*p*-Value	N	Hazard Ratio	*p*-Value	N	Hazard Ratio	*p*-Value
**SEX**												
Male	246	1.5 (0.96–2.35)	0.07	210	1.48 (1–2.21)	0.051	249	1.79 (1.25–2.58)	0.0014	244	1.76 (0.99–3.14)	0.05
Female	118	1.51 (0.84–2.71)	0.17	106	1.84 (1.01–3.37)	0.044	121	1.71 (1.02–2.87)	0.04	118	2.21 (1.02–4.8)	0.04
**STAGE**												
I	170	1.33 (0.72–2.44)	0.36	153	1.39 (0.81–2.4)	0.23	171	1.49 (0.91–2.46)	0.11	168	1.66 (0.68–4.06)	0.27
I+II	253	1.18 (0.73–1.9)	0.49	228	1.42 (0.94–2.17)	0.097	256	1.44 (0.98–2.1)	0.062	251	1.73 (0.86–3.48)	0.12
II	83	1.33 (0.6–2.92)	0.48	75	1.1 (0.57–2.11)	0.78	85	1.31 (0.73–2.36)	0.36	83	1.34 (0.45–4)	0.6
II+III	166	1.45 (0.9–2.32)	0.12	145	1.26 (0.81–1.97)	0.3	170	1.38 (0.93–2.06)	0.11	166	1.43 (0.79–2.6)	0.24
III	83	1.54 (0.85–2.78)	0.15	70	1.35 (0.73–2.49)	0.33	85	1.32 (0.76–2.28)	0.32	83	1.71 (0.84–3.48)	0.13
III+IV	87	1.26 (0.71–2.23)	0.42	70	1.35 (0.73–2.49)	0.33	90	1.36 (0.8–2.31)	0.26	87	1.47 (0.74–2.92)	0.27
IV	4	-	-	0	-	-	5	-	-	3	-	-
**GRADE**												
I	55	2.34 (0.89–6.15)	0.079	45	0.89(0.33–2.41)	0.82	55	2.07 (0.93–4.61)	0.071	55	2.66 (0.77–9.25)	0.11
II	174	1.99 (1.17–3.36)	0.0094	149	1.84 (1.12–3.01)	0.014	177	2.38 (1.52–3.72)	0.000097	171	2.92 (1.44–5.89)	0.0018
III	118	1.23 (0.68–2.25)	0.49	107	1.53 (0.89–2.62)	0.12	121	1.41 (0.86–2.32)	0.18	119	1.08 (0.51–2.31)	0.84
IV	12	-	-	11	-	-	12	-	-	12	-	-
**AJCC_T**												
I	180	1.31 (0.73–2.34)	0.36	160	1.58 (0.93–2.69)	0.09	181	1.55 (0.95–2.52)	0.075	178	1.59 (0.71–3.6)	0.26
II	90	1.38 (0.66–2.87)	0.39	80	1.12 (0.6–2.09)	0.72	93	1.3 (0.75–2.24)	0.35	91	1.4 (0.54–3.64)	0.49
III	78	1.53 (0.83–2.81)	0.17	67	1.2 (0.64–2.26)	0.57	80	1.36 (0.77–2.4)	0.29	77	1.56 (0.75–3.25)	0.23
IV	13	-	-	6	-	-	13	-	-	13	-	-
**Vascular invasion**												
None	203	1.27 (0.76–2.13)	0.35	175	1.15 (0.71–1.86)	0.57	205	1.42 (0.91–2.22)	0.12	201	1.52 (0.74–3.09)	0.25
Micro	90	1.24 (0.57–2.67)	0.59	82	1.2 (0.64–2.25)	0.57	92	1.59 (0.9–2.81)	0.11	90	0.9 (0.3–2.68)	0.85
Macro	16	-	-	14	-	-	16	-	-	14	-	-
**RACE**												
White	181	1.57 (0.99–2.48)	0.055	147	1.46 (0.93–2.3)	0.1	184	1.85 (1.24–2.76)	0.0021	179	2.05 (1.15–3.64)	0.013
Asian	155	2.99 (1.56–5.71)	0.00052	145	1.74 (1.04–2.89)	0.032	157	1.97 (1.22–3.18)	0.0047	154	3.56 (1.49–8.54)	0.0024
**Alcohol consumption**												
Yes	115	1.91 (1.01–3.62)	0.043	99	2.26 (1.24–4.13)	0.0063	117	2.65 (1.55–4.54)	0.00023	117	2.29 (1.1–4.76)	0.023
None	202	1.4 (0.88–2.24)	0.15	183	1.26 (0.81–1.96)	0.31	205	1.44 (0.97–2.16)	0.071	199	1.76 (0.94–3.31)	0.075
**Sorafenib treatment**												
Treated	29	1.42 (0.44–4.63)	0.56	22	3.34 (1.12–9.96)	0.023	30	1.77 (0.78–3.98)	0.16	30	1.62 (0.5–5.24)	0.42
**Hepatitis virus**												
Yes	150	0.82 (0.43–1.56)	0.54	99	2.26 (1.24–4.13)	0.0063	117	2.65 (1.55–4.54)	0.00023	151	1.1 (0.49–2.5)	0.81
None	167	2.79 (1.68–4.62)	0.000037	183	1.26 (0.81–1.96)	0.31	205	1.44 (0.97–2.16)	0.071	165	4.22 (2.17–8.23)	0.0000057

**Table 2:** Correlation between CBX1 expression and DNA methylation in LIHC.

Probe	Chr	Position	Average of Cancer Sample	Average of Normal Sample	*p*-Value
**cg24458315**	chr17	48,071,045	0.84	0.88	*p* < 0.001
**cg26932693**	chr17	48,075,087	0.92	0.94	0.0012
**cg21215337**	chr17	48,098,755	0.91	0.93	*p* < 0.001
**cg18929316**	chr17	48,099,486	0.82	0.86	*p* < 0.001
**cg06150642**	chr17	48,100,857	0.02	0.02	0.0025
**cg20440414**	chr17	48,100,984	0.02	0.03	0.46
**cg11194725**	chr17	48,100,998	0.03	0.03	0.014
**cg12245530**	chr17	48,101,256	0.02	0.01	0.076
**cg11729481**	chr17	48,101,375	0.02	0.02	0.96
**cg17778721**	chr17	48,101,383	0.04	0.04	0.59
**cg04864609**	chr17	48,101,553	0.05	0.04	*p* < 0.001
**cg02835499**	chr17	48,101,556	0.03	0.02	*p* < 0.001
**cg21511817**	chr17	48,101,565	0.02	0.01	0.0076
**cg13342109**	chr17	48,101,569	0.02	0.02	0.72
**cg01553295**	chr17	48,101,633	0.05	0.05	0.86
**cg01544580**	chr17	48,102,907	0.59	0.6	0.54

**Table 3:** miRNA associated with CBX1.

Gene	miRNA	
**CBX1**	hsa-let-7a-5p	hsa-miR-132-3p
	hsa-let-7b-5p	hsa-miR-141-3p
	hsa-let-7c-5p	hsa-miR-126-5p
	hsa-miR-15a-5p	hsa-miR-185-5p
	hsa-miR-24-3p	hsa-miR-200c-3p
	hsa-miR-26a-5p	hsa-miR-155-5p
	hsa-miR-26b-5p	hsa-miR-29c-3p
	hsa-miR-29a-3p	hsa-miR-200a-3p
	hsa-miR-92a-3p	hsa-miR-429
	hsa-miR-96-5p	hsa-miR-494-3p
	hsa-miR-29b-3p	hsa-miR-519d-3p
	hsa-miR-34a-5p	hsa-miR-542-3p
	hsa-miR-212-3p	hsa-miR-297
	hsa-miR-222-3p	hsa-miR-192-3p
	hsa-miR-200b-3p	hsa-miR-145-3p
	hsa-miR-1-3p	hsa-miR-129-2-3p
	hsa-miR-124-3p	hsa-miR-212-5p

## Data Availability

All data are available upon reasonable request to the corresponding author.

## Supplementary Materials

The following supporting information can be downloaded at https://www.mdpi.com/article/10.3390/medicina61060983/s1, Table S1: Construction of lncRNA and circRNA Networks for CBX1-Associated miRNAs in LIHC, Table S2: Correlation of ceRNAs Associated with CBX1 in LIHC.
